# Application of a Physiologically Based Pharmacokinetic Approach to Predict Theophylline Pharmacokinetics Using Virtual Non-Pregnant, Pregnant, Fetal, Breast-Feeding, and Neonatal Populations

**DOI:** 10.3389/fped.2022.840710

**Published:** 2022-05-12

**Authors:** Khaled Abduljalil, Iain Gardner, Masoud Jamei

**Affiliations:** Certara UK Limited (Simcyp Division), Sheffield, United Kingdom

**Keywords:** theophylline, pregnancy, feto-placenta, lactation, preterm, PBPK model, breastfeeding, infant dose

## Abstract

Perinatal pharmacology is influenced by a myriad of physiological variables that are changing dynamically. The influence of these covariates has not been assessed systemically. The objective of this work was to use theophylline as a model drug and to predict its pharmacokinetics before, during (including prediction of the umbilical cord level), and after pregnancy as well as in milk (after single and multiple doses) and in neonates using a physiological-based pharmacokinetic (PBPK) model. Neonatal theophylline exposure from milk consumption was projected in both normal term and preterm subjects. Predicted infant daily doses were calculated using theophylline average and maximum concentration in the milk as well as an estimate of milk consumption. Predicted concentrations and parameters from the PBPK model were compared to the observed data. PBPK predicted theophylline concentrations in non-pregnant and pregnant populations at different gestational weeks were within 2-fold of the observations and the observed concentrations fell within the 5th−95th prediction interval from the PBPK simulations. The PBPK model predicted an average cord-to-maternal plasma ratio of 1.0, which also agrees well with experimental observations. Predicted postpartum theophylline concentration profiles in milk were also in good agreement with observations with a predicted milk-to-plasma ratio of 0.68. For an infant of 2 kg consuming 150 ml of milk per day, the lactation model predicted a relative infant dose (RID) of 12 and 17% using predicted average (C_avg,ss_) and maximum (C_max,ss_) concentration in milk at steady state. The maximum RID of 17% corresponds to an absolute infant daily dose of 1.4 ± 0.5 mg/kg/day. This dose, when administered as 0.233 mg/kg every 4 h, to resemble breastfeeding frequency, resulted in plasma concentrations as high as 3.9 (1.9–6.8) mg/L and 2.8 (1.3–5.3) (5th−95th percentiles) on day 7 in preterm (32 GW) and full-term neonatal populations.

## Introduction

Pharmacokinetics (PK) are typically influenced by a variety of physiological variables and also can be altered in different pathological states (1, 2). During the perinatal period, drug PK can be affected by a variety of time-varying physiological parameters in the mother and the unborn fetus (3). Immediately after birth few physiological parameters are reverting to the pre-pregnancy status and can affect the maternal drug exposure (4). For many drugs, the impacts of these changes are minimal with most of the PK parameters being within the pre-pregnancy range (5, 6). Drug exposure to neonates after birth may happen during breastfeeding, assuming the drug reaches the milk after maternal intake. The amount of drug delivered to the breastfed neonate varies with variability in maternal physiology and milk composition as well as the breastfeeding style, i.e., frequency and fed amounts (7, 8). Once the drug reaches the neonatal gut or systemic circulation, the exposure in neonates is influenced by the maturation of the drug absorption and disposition processes that are known to vary with the age of the newborn (9–11).

The impact of physiological changes during pregnancy on drug disposition has not always been thoroughly assessed in clinical studies. This leaves open the question of how and to what extent physiological changes can affect the PK of a drug during pregnancy and if knowledge of the physiological changes that occur during pregnancy can be used to provide some insight into potential PK alterations during pregnancy. Information on the expected alteration of drug PK during the perinatal period can be used to guide the initial prescription strategies to protect both the mother and the neonate by aiding the selection of the right dose for the right patient at the right time (12, 13). During breastfeeding, there is a risk of neonatal drug exposure *via* breast milk following maternal drug intake. Milk is a complex fluid, with pH, fat, and protein levels that change over time. The composition of milk and the physicochemical properties of drugs largely determine the extent to which drugs are excreted into the milk (14). The ability to predict neonatal exposure to drugs *via* breast milk (particularly those that are potentially hazardous to neonates) would also be useful in a clinical setting.

Physiologically-based-pharmacokinetic (PBPK) modeling has been widely used to investigate the influence of physiological changes in different subjects or in specific populations on drug disposition (15, 16). The application of PBPK models to predict drug exposure in pregnant women is increasing due to its mechanistic nature. This allows the inclusion of gestational age-related changes in physiological parameters together with information on the physicochemical properties, *in vitro* disposition information (binding, metabolism, permeability, solubility, etc.), and human PK of the drug to be considered in the PBPK model (12). To date, the clinical applications of the PBPK model to predict drug exposure in milk are still very limited. The aim of this work is to develop a PBPK model to describe the pharmacokinetics of theophylline before, during, and after pregnancy, in breast milk, and in neonates. Theophylline is commonly used to treat asthma and apnea of prematurity and was selected as a model drug for this exercise due to the availability of PK data from different perinatal periods.

## Materials and Methods

### Workflow

**Figure 1:**
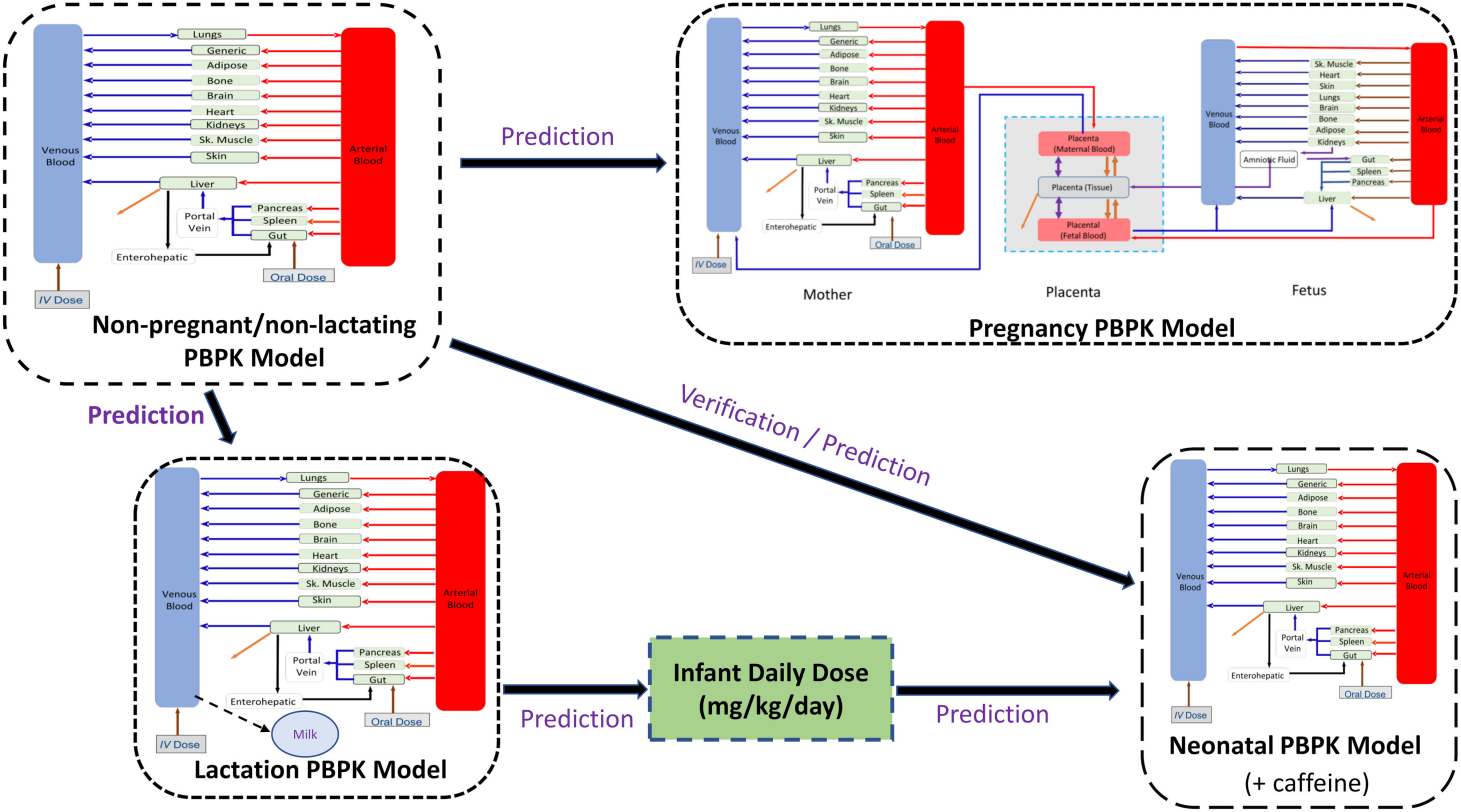
Workflow of the implemented perinatal theophylline physiological-based pharmacokinetic (PBPK) model. The neonatal model includes caffeine PBPK as a formed metabolite.

For all predictions of theophylline kinetics in different populations, the Simcyp Simulator (V21) was used. The workflow of the PBPK model implementation was as follows. Firstly, simulations were performed to predict the theophylline PK in non-pregnant subjects. Secondly, the developed theophylline PBPK model in non-pregnant subjects was used to predict the theophylline PK during pregnancy by applying gestational-dependent changes in the physiological parameters of the mother and the fetus. Thirdly, the PBPK model was coupled with a lactation model (14) to predict the drug exposure in maternal plasma and milk (8). Finally, the predicted infant daily dose from the lactation model was used as a dose input for the neonatal PBPK model in neonatal subjects of different ages by accounting for neonatal age-dependent physiology changes (17). This workflow is depicted in Figure 1. The results from all simulations were compared to observed clinical data. A total of 20 trials were used in each executed simulation using the reported sample size for each trial to the derived parameters e.g., AUC, Cmax, etc., are reasonable estimates of the parameters and their associated variability in a population. If a clinical study used < 10 subjects, we executed the simulation for 10 subjects in 20 trials (200 subjects) to get a better picture of the variability.

### Model Building

The input parameters for the theophylline PBPK model are provided in the Supplementary Table 1. The parameters used in the theophylline PBPK model are based on a previously published theophylline PBPK model (3) that was used to simulate theophylline PK in non-pregnant and pregnant women without considering the fetoplacental model or CYP2E1 changes during pregnancy. The absorption was modeled using a first-order absorption model. In the current work, a mechanistic model of oral absorption of theophylline was used with the permeability in different segments of the intestine being predicted from physicochemical properties using a mechanistic permeability approach (18).

Depending on the clinical study the theophylline was dosed in the PBPK model either as a solution or a tablet. After oral administration of theophylline in adult subjects, the bioavailability is ~100% from uncoated tablet and liquid formulations (19). When tablets were used in the clinical study the solid formulation option was chosen for the PBPK model with dissolution being described using a diffusion layer model (20) with an intrinsic solubility value for theophylline being calculated from the melting point and lipophilicity of the drug [273°C; (21)] (22). The distribution of theophylline into the tissues was described using a full-body PBPK model with tissue partition coefficients (Kps) being predicted according to Rodgers and Rowland (23) with a global tissue scalar of 1.2 to recover reported data after an intravenous dose (24). The elimination of theophylline was described using metabolism (~85% of systemic clearance) and renal clearance data (~15% of systemic clearance) (see Supplementary Table 1) for a list of input parameters in the PBPK model). The metabolism of theophylline in non-pregnant subjects was mainly by CYP 1A2 (~88% of hepatic metabolism) and CYP2E1 (~12% of hepatic metabolism) with minor contributions (<1%) from CYP2D6 and CYP3A4). Assignment of the contribution of individual CYP isozymes to the metabolism of theophylline was made based on published data (25, 26). The adequacy of these parameters to predict theophylline PK in non-pregnant subjects was assessed by comparison with observed data in non-pregnant populations after intravenous and oral administrations. The compound PBPK model was then used to simulate theophylline PK in the pregnancy, lactation, and neonatal PBPK models. In these simulations, the input parameters for theophylline were not modified with the exception of the inclusion of a metabolic pathway resulting in the formation of caffeine in neonatal subjects that is not observed in adult subjects (27, 28) (Supplementary Table 1). In addition, a first-order absorption model was used in the preterm subjects as the mechanistic absorption model used in the other populations has not been implemented in the software for preterm subjects due to a paucity of appropriate physiological gut data to parametrize the more complex absorption model in preterm subjects. Other physiological changes in the PBPK model were accounted for in pregnant women and neonatal subjects (see following sections for details).

### Theophylline PK in Non-Pregnant Population

The following virtual trial settings were used for non-pregnant subjects after either intravenous or oral administrations:

*Trial design NP1* (model building)*:* Single intravenous infusion of 4.5 mg/kg theophylline administered over 30 min (24); 20 trials of 14 (0% women) subjects aged 19–35 years.

*Trial design NP2:* Single intravenous infusion of 7.3 mg/kg theophylline administered over 30 min (19); 20 trials of 20 (50% women) subjects aged 24–57 years.

*Trial design NP3:* Single intravenous infusion of 240 mg theophylline administered over 45 min (29); 20 trials of 10 (100% women) subjects aged 22–26 years.

*Trial design NP4:* Single intravenous infusion of 151.2 mg theophylline administered over 20 min (30); 20 trials of 13 (0% women) subjects aged 20–39 years.

*Trial design NP5:* Single oral doses of 3.4 mg/kg theophylline (31); 20 trials of 10 (56% women) subjects aged 18–36 years.

*Trial design NP6:* Single oral dose of 5 mg/kg theophylline solution (32); 20 trials of 12 subjects (0% women) aged 23–39 years.

*Trial design NP7:* Single oral dose of 5 mg/kg theophylline solution (33); 20 trials of 10 (50% women) subjects aged 22–35 years.

*Trial design NP8:* Single oral dose of 7.6 mg/kg theophylline (19); 20 trials with 10 (50% women) subjects aged 22–57 years in each trial.

*Trial design NP9:* Single ascending oral dose of 125, 250, 375, and 500 mg theophylline tablet (34); 20 trials of 10 (50% women) subjects aged 22–35 years in each trial.

### Theophylline PK During Pregnancy

The changes in maternal physiology during pregnancy have been described in detail previously (3). The main physiological changes affecting the clearance of theophylline during pregnancy are the changes in CYP1A2 and CYP2E1 activity and renal GFR during pregnancy. These changes were described in the PBPK model using the following functions:


$$\begin{array}{lll} {CYP1A2}_{preg} & = & {CYP1A2}_{\left(0\right)}\;\left(1-0.02552\;GW+0.0002\;GW^{2}\right)\\ {CYP2E1}_{preg} & = & {CYP2E1}_{\left(0\right)}\;\left(1+0.012\;GW+0.0002\;GW^{2}\right)\\ GFR_{pred}\left(\frac{mL}{\min}\right) & = & GFR_{\left(0\right)}\;\left(1+\;0.028392\;GW-0.000502\;GW^{2}\right) \end{array}$$

where *CYP*1*A*2_(0)_, *CYP*2*E*1_(0)_, and *GFR*_(0)_ are the baseline values in non-pregnant women. Values for individual subjects *i*, are generated from a mean value and %CV using lognormal distribution, GW is the gestational week. These equations, except CYP2E1, have been described previously (3, 35). The change of CYP2E1 activity during pregnancy used in this study was derived based on the difference in the longitudinal *unbound* oral clearance of theophylline observed during pregnancy and the predicted *unbound* clearance from the PBPK model without CYP2E1 changes being incorporated (Supplementary Figure 1). Due to the absence of systemic clearance at different gestational weeks, the oral clearance was used as the bioavailability of theophylline was reported to be complete, i.e., F = 1 (19). Exposure in the fetus was simulated to occur *via* a placental permeability-limited model as described previously (36). In the current work, the fetal model was extended to 14 compartments representing various fetal tissues and linked to the maternal full-PBPK model *via* the placenta, which in turn was represented by three compartments (Figure 1). Growth of the fetus and fetal tissues, tissue blood flows, and binding proteins were all dynamic within the model according to previously published relationships (37–40). Physiological changes to the placenta, including its size and blood flow on the maternal and fetal sides, were also included in the model (35, 40). More details on the fetoplacental model assumptions and application have been described elsewhere (41).

This maternal-fetal model allows the prediction of fetal exposure. A value for theophylline transplacental clearance (CL_PD_) obtained from an *ex vivo* experiment of 2.59 mL/min/cotyledon (42) was included in the PBPK model to predict umbilical cord exposure. The *in vitro* value was scaled to give a CL_PD_ value in (L/h/g tissue) as described below using the reported cotyledon weight of 22 g reported in the same experiment (42):


$$\begin{array}{l} {Placenta\;CL}_{PD}\;=\frac{2.59\;\left(mL/\min\right)}{22\;\left(g\right)}\;60/1000\\ \;=0.0071\;L/h/g\;tissue \end{array}$$

This CL_PD_ of *0.0071 L/h/g tissue* was used as a model input parameter to parametrize the passive diffusion clearances on both sides of the placenta assuming a placental density of 1 g/ml. In addition, to predict the amniotic exposure of theophylline, the fetal renal clearance (*fetal CL*_*R*_) was calculated based on fetal GFR of 4.9 ml/min (43) with reference to a typical adult GFR value of 121 mL/min (44) and the adult theophylline renal clearance of 0.31 L/h (see Supplementary Table 1) according to the following equation


$$\begin{array}{l} {fetal\;CL}_{R}\left(L/h/kg\right)=\left(\frac{\;Adult\;CL_{R}\;\left(L/h\right)}{Fetal\;Bodyweight\left(Kg\right)}\right)\\ \;\left(\frac{fetal\;GFR\;\left(mL/min\right)\;}{Adult\;GFR\;\left(mL/min\right)\;}\right) \end{array}$$

Clearances between the fetal tissue and amniotic fluid, as well as fetal swallowing, were accounted for in the fetal PBPK model as described previously (41). The full list of the model input parameters is available in Supplementary Table 1.

The following trial designs were set for model prediction during pregnancy to match the clinical studies after oral administration of theophylline:

*Trial design P1:* Multiple oral doses of 259 mg theophylline for 5 days (45); 20 trials of 10 pregnant women aged 19–31 years at 13–19 GWs.

*Trial design P2:* Multiple oral doses of 259 mg theophylline for 5 days (45); 20 trials of 10 pregnant women aged 19–31 years at 23–28 GWs.

*Trial design P3:* Multiple oral doses of 259 mg theophylline for 5 days (45); 20 trials of 10 pregnant women aged 19–31 years at 34–39 GWs.

*Trial design P4:* Multiple oral doses of 259 mg theophylline for 5 days (46); 20 trials of 10 pregnant women aged 20–31 years at 37–40 GWs.

*Trial design P5:* Multiple oral doses of 160 mg theophylline every 6 h for 3 days (47); 20 trials of 10 pregnant women aged 19–31 years at 34–39 GWs.

*Trial design P6:* Multiple oral doses of 100 mg theophylline every 12 h for 3 days (48); 20 trials of 10 pregnant women aged 18–45 years at 40 GWs.

### Theophylline PK During Lactation

Due to an absence of information on the milk composition of the nursing mothers included in the clinical studies, two empirical models (I and II; see below for detailed equations) were used to predict the theophylline milk-to-plasma (M/P) ratio assuming a mature milk composition (8) and the average value of milk:plasma ratio (M/P) was used in the simulations (see Discussion section).

Model I (14, 49):


$$M/P\;=\;\frac{fu_{p}fu_{p}^{un}}{fu_{mk\;}^{un}}\;\frac{1}{fu_{mk}\left(\frac{1}{1+\;f_{fat}\;(fu_{mk}Papp_{milk}-1)}\right)}$$

Model II (14):


$$\begin{array}{l} \ln M/P=\;-0.405\;+\;9.36\;\ln\left(Mu/Pu\right)-\;0.69\;\ln{fu}_{p}-1.54\;InK \end{array}$$

where,


$$\begin{array}{l} K\;=\left(\left(1-{\;f}_{fat}\right)/{\;fu}_{mk}\right)+{\;f}_{fat}\;.\;Papp_{milk}\\ Papp_{milk}=10^{\left(-0.88+1.29\;LogD_{7.2}\right)} \end{array}$$

*fu*_*p*_ is the individualized unbound fraction of the drug in the maternal plasma, *f*_*fat*_ is the fractional volumes of fat components in the milk, sampled from a population mean of 6.2 g/100 ml of milk and distribution (33% CV) using a lognormal distribution. *LogD*_7.2_ is the apparent milk fat-to-skimmed milk partition at pH 7.2 (14). This value is predicted from the theophylline octanol-to-water partitioning ratio (*LogP*_*o*:*w*_) accounting for ionization at pH = 7.2. *fu*_*mk*_ is the individualized unbound fraction of the drug in the milk calculated using the following equation (50):


$$\begin{array}{l} fu_{mk}=\frac{{fu_{p}}^{0.448}}{\;0.000694^{0.448}+\;{fu_{p}}^{0.448}} \end{array}$$

*Mu*/*Pu*, is the ratio of the unionized fraction of the drug in plasma, $f_{p}^{un}$, to the unionized fraction of the drug in milk, $f_{mk}^{un}$. $f_{p}^{un}$, is calculated using the compound pKa(s) and the plasma pH. According to the following equations:


$$\begin{array}{l} f_{p}^{un}=\;\frac{1}{{1+10}^{\left(pKa2-pKa1\right)}+10^{\left(pH_{plasma}-pKa1\right)}\;+10^{\left(pKa2-pH_{plasma}\right)}}\\ f_{mk}^{un}=\;\frac{1}{{1+10}^{\left(pKa2-pKa1\right)}+10^{\left(pH_{Milk}-pKa1\right)}\;+10^{\left(pKa2-pH_{Milk}\right)}} \end{array}$$

Where the pH of the milk is a physiological parameter (milk pH = 7.0) (8).

Using the average milk to plasma ratio, the milk level of theophylline after single and multiple dosing in the mother was simulated. Predicted infant daily doses were calculated using the predicted theophylline average (C_avg,ss_) and maximum (C_max,ss_) concentration in milk at a steady state.

*Trial design L1:* Multiple oral doses of 259 mg theophylline for 5 days (45); 20 trials of 10 nursing mothers aged 19–31 years in each trial.

*Trial design L2:* Single intravenous dose of 4.25 mg/kg infused over 20 min (51); 20 trials of 10 nursing mothers aged 19–31 years in each trial.

*Trial design L3:* Oral doses of 300 mg theophylline followed by 200 mg after 4 h (52); 20 trials of 12 nursing mothers aged 20–40 years old.

### Theophylline PK in Neonates

For assessment of theophylline neonatal exposure from milk, the calculated infant daily dose was used as an input for the neonatal PBPK model. Simulations were conducted in both full-term and preterm neonatal subjects. The physiology of the preterm PBPK model includes age-dependent changes in physiology, including parameters relevant to theophylline elimination such as renal function, and CYP1A2 ontogeny (53). The ontogeny of CYP2E1 has not been quantified in preterm individuals so the ontogeny in preterm subjects was assumed to be the same as in the full-term subjects (54). Therefore, the following equations were used to describe the age-related changes in theophylline clearance:


$$\begin{array}{l} \;GFR_{preterm}\;\left(mL/\min\right)=\;121\left(\frac{PMA^{3.4}}{0.923^{3.4}+PMA^{3.4}}\right)\\ {\;(\frac{weight}{70})}^{0.75}\\ {CYP1A2}_{preterm}\;\left(fraction\;of\;adult\right)=\;0.81+PMA^{5.2}\\ {CYP2E1}_{preterm}\;\left(fraction\;of\;adult\right)=\;\frac{0.99\;x\;{\left(PNA/52\right)}^{0.5}}{{0.23^{0.5}+\left(PNA/52\right)}^{0.5}} \end{array}$$

where PMA is the postmenstrual age in years, and PNA is the postnatal age in weeks converted to years *via* dividing by 52 weeks.

Theophylline undergoes an additional metabolic process in preterm neonates resulting in the formation of caffeine (27). This metabolic pathway does not occur in adults and the pathway is therefore not included in the adult model. The pathway leading to caffeine formation was accounted for in the preterm neonatal PBPK model as described below with no further ontogeny of the pathway being considered in the liver. A previously verified caffeine PBPK model (53) was included in the preterm neonatal model as a metabolite of theophylline and was used to optimize the intrinsic clearance of theophylline to caffeine by comparison of the simulated with the observed caffeine levels in preterm neonates after intravenous (28) and oral administration (55) of theophylline. A scaling factor of 20-fold for the conversion of theophylline to caffeine in the gut was required to describe the observed exposure of theophylline, but also the formed caffeine checked during the exercise, after oral administration. Sensitivity analysis for this intestinal metabolism scalar is given in Supplementary Figure 4. The caffeine metabolite model was retained in the PBPK model for all neonatal simulations. A list of preterm PBPK model inputs for theophylline and caffeine is given in Supplementary Tables 1, 2. To account for fast developmental changes in the preterm physiology, the time-varying covariates option within the Simulator was used (56).

The following simulations were conducted for the neonatal model building and performance verification:

*Trial design N1* (model building)*:* An intravenous loading dose of 5.5 mg/kg theophylline infused over 20 min followed by multiple intravenous doses every 12 h of 1.1 mg/kg infused over 1 h for 7 days (28); 20 trials of 10 (20% women) preterm neonates aged 0–3 postnatal weeks and their gestational weeks ranged between 27 and 32 weeks. The systemic concentration profiles were followed for 14 days from the first dose.

*Trial design N 2:* a single IV bolus of 1.2 mg/kg of theophylline to produce the same initial plasma concentration in the neonatal PBPK model that was observed in the umbilical cord plasma at birth (48); 20 trials of 10 full-term neonates at birth (0 h PNA).

*Trial design N 3:* a single IV infusion of 4 mg/kg theophylline for 20 min (57); 20 trials of 10 (50% women) preterm neonates aged 3–15 days and GWs of 27.5 weeks.

*Trial design N 4* (model building): a loading oral dose of 5 mg/kg theophylline then 1.25mg/kg orally every 6 h (55); 20 trials of 14 (50% women) preterm neonates aged 85 h and their gestational weeks ranged between 25 and 34 weeks.

*Trial design N5:* Single oral dose of 5.6 mg/kg theophylline solution (58); 20 trials of 10 (50% women) preterm neonates aged 0–4 postnatal weeks and their gestational weeks ranged between 29 and 36 weeks.

*Trial design N6*: a loading oral dose of 5 mg/kg theophylline followed by 8 doses of 2.3 mg/kg every 12 h [subject Sch in (58)]; 20 trials of 10 (50% women) preterm neonates aged 2–28 days and 34 gestational weeks. A similar experimental design was used but with 7 doses of 2 mg/kg every 12 h [subject C in (58)].

*Trial design N7:* Multiple oral doses of 1.25 mg/kg theophylline every 6 h (27); 20 trials of 10 (30% women) preterm neonates aged 1–9 days with their gestational weeks ranging between 26 and 33 weeks.

*Trial design N8:* A loading dose of 5 mg/kg theophylline infused over 30 min followed by 1 h-infusion of 1.1 mg/kg/12 h in 28 (A), 32 (B), and 38 (C) GWs neonates at birth. This replicates the study design reported by Bonati et al. (28).

*Trial design N9:* Multiple oral doses of the predicted average infant daily dose divided into 6 daily doses. This dosing pattern resembles the frequency of feeding for breastfed babies. Dosing was repeated for 14 consecutive days; 20 trials of 20 (50% women) neonates at birth, either 28, 32, or 38 GWs (three scenarios). In this trial, a single intravenous loading dose of 4.2 mg/kg (for a 28 GWs group), 4.5 mg/kg (for a 32 GWs group), and 4.8 mg/kg (for a 38 GWs group) was administered over 10 s to produce an initial systemic concentration of 10 mg/L, the same concentration as was observed in the cord plasma at birth.

*Trial design N10:* Multiple oral doses of the predicted maximum infant daily dose divided into 6 daily doses. This dosing pattern resembles the frequency of feeding for breastfed babies. Dosing was repeated for 7 14 consecutive days; 20 trials of 20 (50% women) neonates at birth, either 28, 32, or 38 GWs (three scenarios). A single intravenous loading dose as described in *Trial design N9* was used here as well to give an initial plasma concentration the same as that observed in the cord plasma at birth.

*Trial design N11:* Same design as in *Trial design N10*, but without any loading dose.

### Assessment Criteria

Dependent on data availability, the predicted PK profiles and/or PK parameters were compared with different sets of clinical observations available in the literature. The PBPK model predictions were considered successful and acceptable if the observed PK profile fell within the 95th and 5th percentile of predicted data and the predicted PK parameters fell within 0.5- to 2-fold of the observed data.

## Results

**Figure 2:**
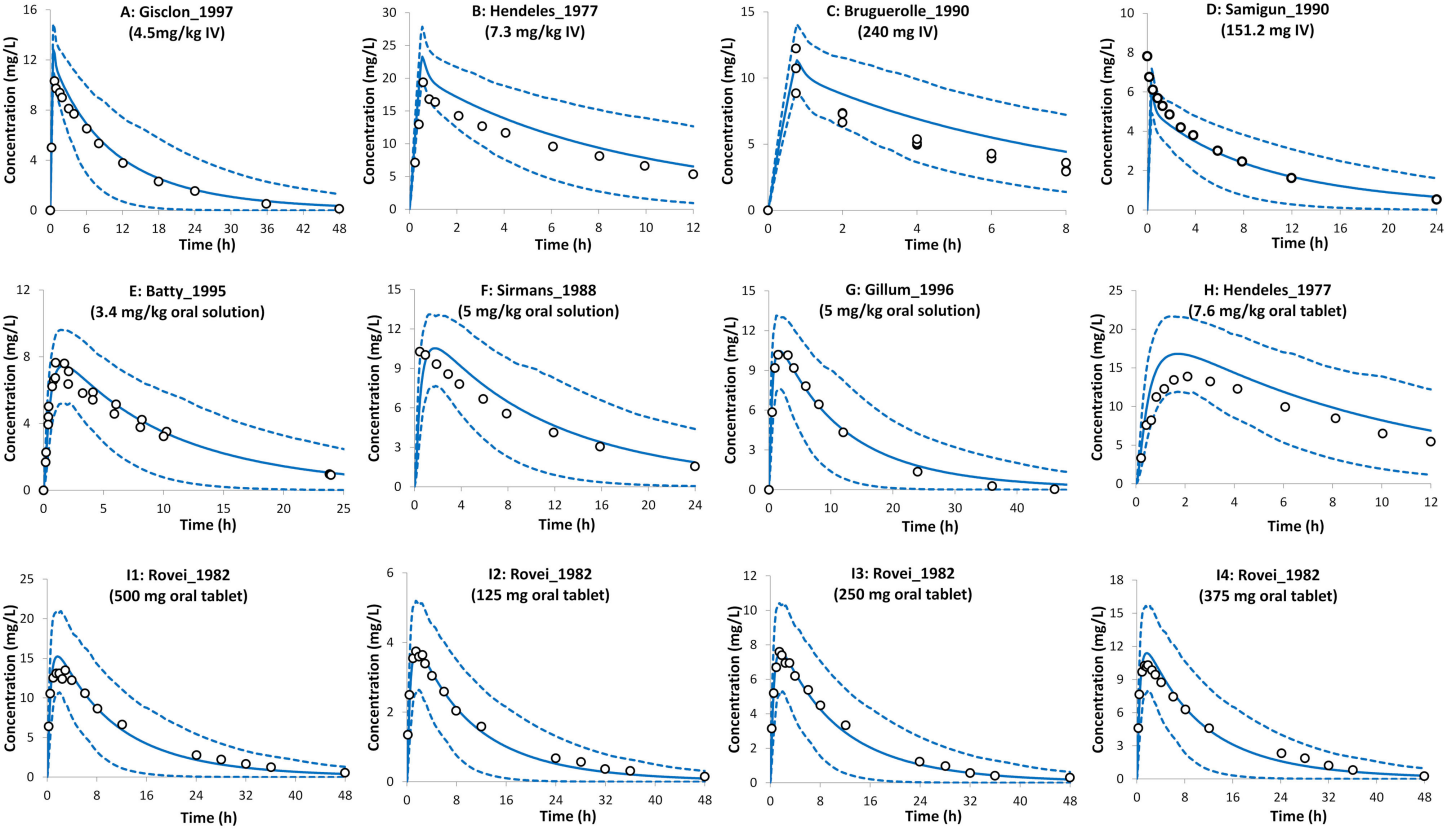
Plasma concentration profiles after intravenous infusion and oral administration in the non-pregnant population. Solid lines, predicted means; Dashed lines, 5th and 95th centiles; Circles, observed means. **(A)** *Trial design NP1* (24), **(B)** *Trial design NP2* (19), **(C)** *Trial design NP3* (29), **(D)** *Trial design NP4* (30), **(E)** *Trial design NP5* (31), **(F)** *Trial design NP6* (32), **(G)** *Trial design NP7* (33), **(H)** *Trial design NP8* (19), and **(I1–I4)** *Trial design NP9* (34). See the Method section for trial settings.

Theophylline simulations for the baseline model in non-pregnant subjects are shown in Figure 2. The PBPK model predictions agreed with the observed mean profiles in different studies after intravenous and oral administrations. The predicted mean concentration profile follows the same shape as the observed mean concentration profiles and the observed data fell within the simulated 5th−95th prediction interval. A comparison of the predicted PK parameters in the non-pregnant population with those available from the clinical studies is shown in Table 1.

**Table 1:** Predicted vs. observed theophylline pharmacokinetics (PK) parameters in pre-pregnant, pregnant, breastfeeding, and neonatal populations.

**Population *N* (%Female)**	**Dose**	**CL (L/h)** ^**a**^			AUC (mg^*^h/L)			**Cmax (mg/L)**		
		**Obs**	**PRED**	**Ratio**	**Obs**	**PRED**	**Ratio**	**Obs**	**PRED**	**Ratio**
Non-Preg 14 (0%)	4.5 mg/kg–30 min INF (24)	2.84 ± 0.62	3.19 ± 1.8	1.12	126 ± 30	144 ± 72	1.14	10.7 ± 1.3	13.5 ± 1.2	1.26
Non-Preg 20 (50%)	7.3 mg/kg–30 min INF (19)	2.53	3.02 (0.7–11)	1.19	184 (95–287)	232 (48–716)	1.26	NA	23.3 (18.2–35.4)	NA
Non-Preg 9 (100%)	240 mg–45 min INF (29)	3.15	2.9 ± 1.6	0.92	76 ± 35	103 ± 48	1.36	10.7	11.3 ± 1.5	1.06
Non-Preg 13 (0%)	151.2 mg–20 min INF (30)	2.8 ± 0.9	3.20 ± 1.80	1.14	NA	60 ± 31	NA	NA	6.01 ± 0.68	NA
Non-Preg 9 (55.6%)	3.4 mg/kg oral solution (31)	2.96	3.2 ± 1.9	1.10	86.12	97.8 ± 48	1.14	NA	7.62 ± 1.2	NA
Non-Preg 12 (0%)	5 mg/kg—oral solution (32)	3.18 ± 0.75	3.4 ± 2.0	1.06	134 ± 34	155 ± 79	1.15	NA	10.3 ± 1.6	NA
Non-Preg 11 (0%)	5 mg/kg—oral solution (33)	3.2 ± 0.7	3.46 ± 2.1	1.08	140 ± 24	152 ± 79	1.09	10.9 ± 1.1	10.7 ± 1.6	0.98
Non-Preg 10 (50%)	6.7 mg/kg oral tablet (19)	3.05	3.1 ± 1.8	1.02	173 (88–283)	231 (54–738)	1.34	15.3 (13–20)	17.2 (11–23.4)	1.12
Non-Preg 8 (50%)	125 mg oral tablet (34)	2.7 (1.6–3.8)	3.3 (0.80–10.8)	1.22	52 (31–94)	48.8 (12.0–157)	0.94	4.1 (3.0–6.7)	3.86 (2.3–6.7)	0.94
Non-Preg 8 (50%)	250 mg oral tablet (34)	2.54 (1.74–2.98)	3.3 (0.80–11)	1.3	106 (69–172)	99 (23–316)	0.93	8.0 (5.0–12.1)	7.74 (4.5–13.34)	0.97
Non-Preg 8 (50%)	375 mg oral tablet (34)	2.6 (1.8–4.0)	3.3 (0.80–10.6)	1.27	161 (75–272)	150 (35–479)	0.93	10.5 (6.7–15.0)	11.7 (6.81–20.1)	1.11
Non-Preg 8 (50%)	500 mg oral tablet (34)	2.54 (1.61–3.16)	3.2 (0.78–10.5)	1.26	210 (136–373)	202 (48–643)	0.96	15.1 (10.7–20.5)	15.6 (9.1–27)	1.03
Preg 13–19 GWs 10 (100%)	259 mg oral solution/12 h (45)	2.61 ± 0.63	2.50 ± 1.3	0.96	99.23	127 ± 57	1.28	NA	14.6 ± 5.0	NA
Preg 23–28 GWs 10 (100%)	259 mg oral solution/12 h (45)	2.85 ± 1.05	2.47 ± 1.3	0.87	90.88	131 ± 58	1.44	NA	14.7 ± 4.8	NA
Preg 34–39 GWs 10 (100%)	259 mg oral solution/12 h (45)	2.1 ± 0.49	2.2 ± 1.0	1.04	123.3	142 ± 59	1.15	NA	15.3 ± 4.7	NA
Lactation 10 (100%)	259 mg oral solution/12 h (45)	2.16 ± 0.81	3.0 ± 1.7	1.39	120	112 ± 55	0.93	NA	13.5 ± 4.7	NA
Lactation 12 (100%)	300 mg followed by 200 mg—oral (52)	NA	3.0 ± 1.7	NA	NA	NA	NA	NA	10.5 ± 1.8	NA
Preterm^*^ 9 (30%) GW: 27–31; PNA: 0–1	5.5 mg/kg i.v followed by 1.1 mg/kg/12 h i.v. (28)	14.5^#^^,^^a^	11.2 ± 3.7^#^ (4.91–25.3)^**^	0.803	NA	118 ± 37 (46–233)^**^	NA	NA	10.9 ± 3.3 (5.0−20)^**^	NA
Preterm^*^ 6 (NA) GW: 27.5; PNA: 0.5–3	4 mg/kg infusion over 20 min (57)	17.6^**^ (12.1 – 25.9)	25.9 ± 1.7 (22–31)^**^	1.47	NA	155 ± 10 (128–184)^**^	NA	NA	9.4 ± 0.24 (8.9–10.4)^**^	NA
Preterm^*^ 15 (NA) GW: 25–34; PNA: 1–2	(NA) (59)	15.4 ± 6.8^#^^,^^a^ (3.3–31.4)	12.5 ± 4.7^#^^,^^a^ (5.0–34)	0.81	NA	99 ± 35 (32–232)^**^	NA	NA	9.3 ± 2.9 (3.7–20)^**^	NA
Preterm^*^ 11 (50%) GW = 32–36; PNA: 0.7–4	5.6 mg/kg /8 h oral Solution (58)	28.3 ± 6.4^a^ (20.1–40.1)	25.9 ± 17.2^a^ (6.9–123)	0.92	NA	100 ± 37 (25–235)^**^	NA	7 (6.4–8)	6.8 (2.8–9.0)	0.90

* *Preterm: GW, gestational age in weeks; PNA, postnatal age in weeks*,

a *preterm clearance unit is mL/h/kg*,

# *blood clearance*,

** *range, NA, not mentioned. For preterm simulations, 50% of simulated subjects were female. For preterm AUC was calculated as the last AUC_tau_*.

**Figure 3:**
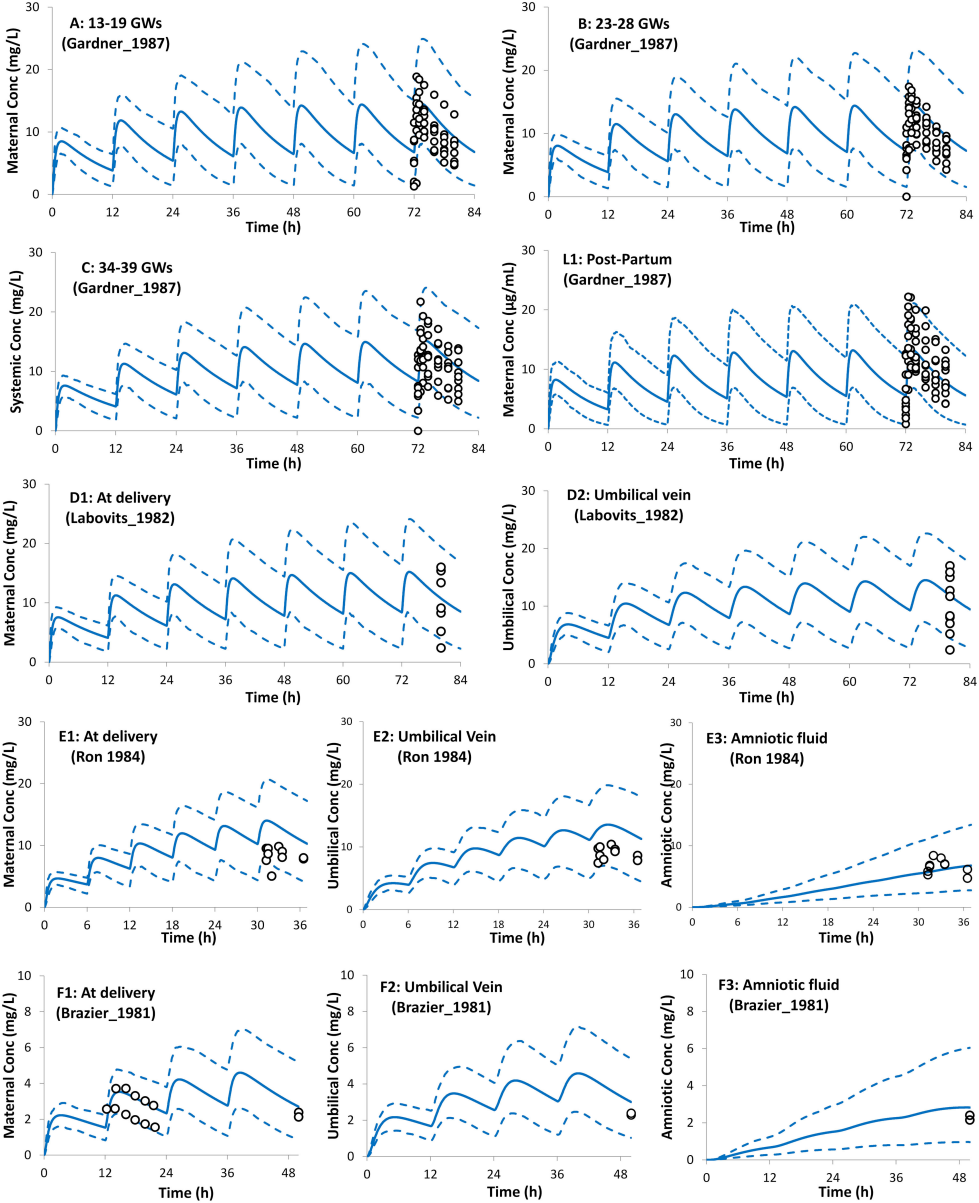
Plasma concentration profiles after multiple oral administration in pregnant population during pregnancy and at delivery. Solid lines, predicted means; Dashed lines, 5th and 95th centiles; Circles, individual observations (open, maternal; filled, umbilical cord). Plots representing the following trials: **(A)** *Trial design P1* (45), **(B)** *Trial design P2* (45), **(C)** *Trial design P3* (45), **(L1)** *Trial design L1* (45) added here for comparison (see lactation section), **(D1,D2)** *Trial design P4* (46), **(E1–E3)** *Trial design P5* (47), and **(F1–F3)** *Trial design P6* (48). See the Method section for trial settings.

The PBPK model predictions during the different trimesters of pregnancy are shown in Figure 3. The predicted data agree with the observed data within the pre-defined success criteria. Limited observed data were available for theophylline exposure during delivery. However, the predicted plasma and umbilical cord concentrations of theophylline during labor agreed with the reported observed concentrations (Figure 3). The model predicted a mean cord-to-plasma AUC ratio of 1 ± 0.1 (range: 0.8–1.3) at a steady state. A comparison of the predicted PK parameters during pregnancy with those available from clinical studies is presented in Table 1.

**Figure 4:**
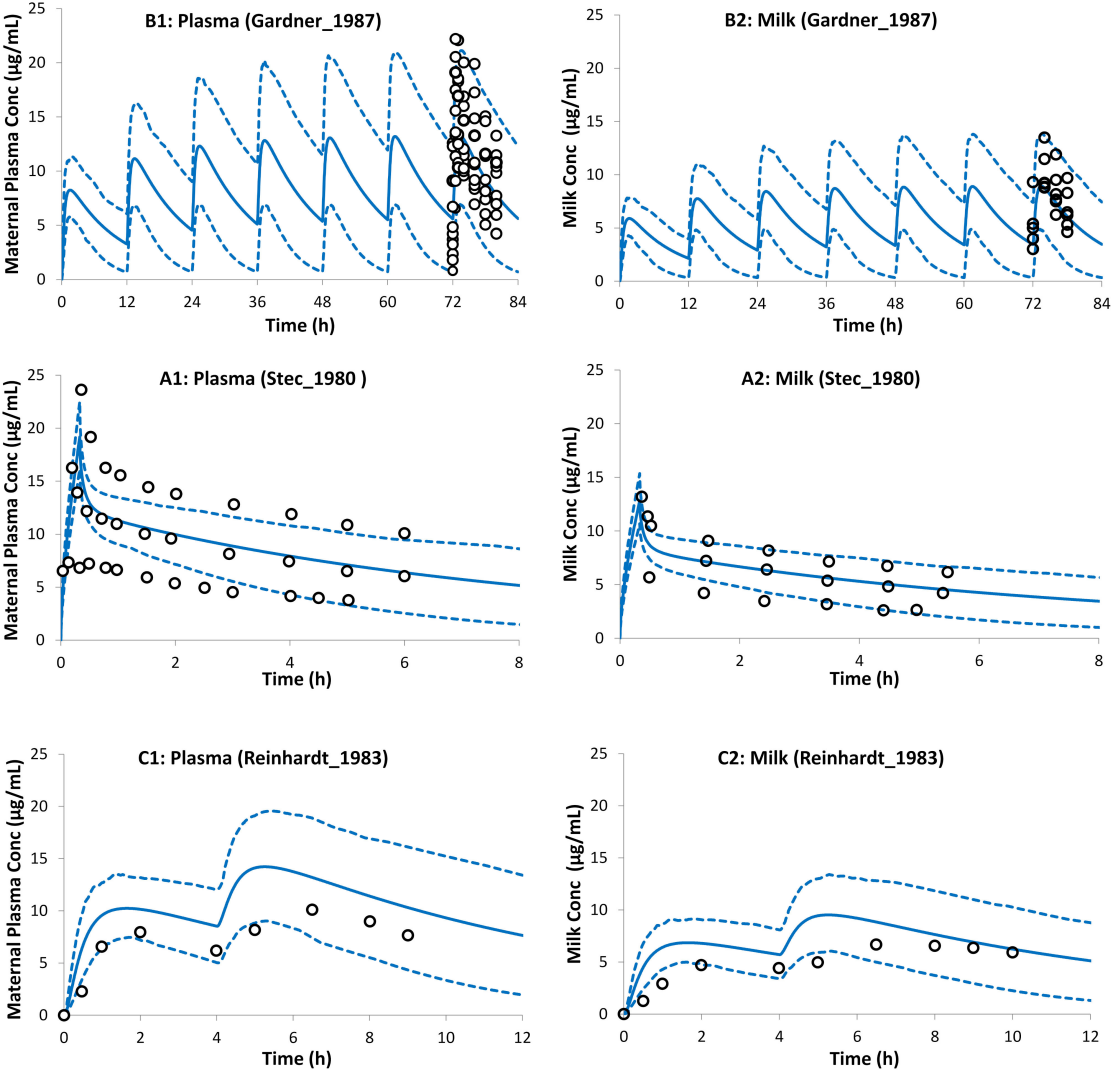
Theophylline concentration profiles in maternal plasma (left) and the milk (right). Milk exposure was predicted using the average predicted M/P ratio from both lactation models (see Method section). Solid lines, predicted means; Dashed lines, 5th and 95th centiles; Circles, individual observations (open, maternal; filled, milk). **(A1,A2)** *Trial design L1* (45), **(B1,B2)** *Trial design L2* (51), and **(C1,C2)** *Trial design L3* (52). See the Method section for trial settings.

Predicted postpartum theophylline concentrations in the maternal plasma were in good agreement with observations (Figure 4). Lactation empirical methods predicted different mean M/P ratios (0.49 for Model I and 0.87 for Model II; see Supplementary Figure 3), hence the mean value (0.68 ± 0.05) of these predicted ratios was used, which resulted in better. agreement with observations (Figure 4). For a preterm infant of 2 kg consuming 150 ml of milk/day, the lactation model predicted a relative infant daily dose (RID) of 12 ± 5% (5th−95th percentiles: 5–20) using milk C_avg,ss_, increasing to a RID of 17 ± 6% (5th−9th percentiles: 9–26) using milk C_max,ss_. These RID values correspond to absolute values of 0.94 ± 0.4 (5th−95th percentiles: 0.4–1.61) mg/kg/day, and 1.4 ± 0.5 (5th−95th percentiles: 0.74–2.1) mg/kg/day for C_avg,ss_ and C_max,ss_ doses, respectively.

**Figure 5:**
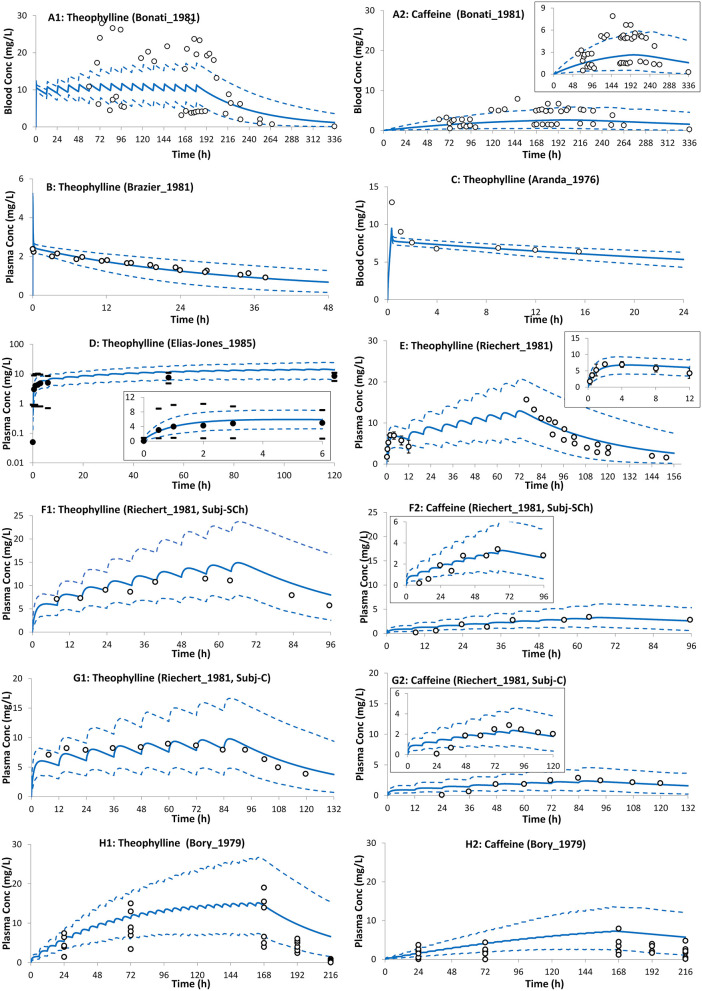
Theophylline (and formed caffeine) concentration profiles in neonates after intravenous **(A–C)** and oral **(D–H)** administration of theophylline. Solid lines, predicted means; Dashed lines, 5th and 95th centiles; closed circles, individual observations; closed circles **(D,E)**, mean; dashes associated with observations in **(D)** represent reported ranges, and bars [**(E)**; till 12 h] represent SD. **(A1,A2)** *Trial design N1* (28), **(B)** *Trial design N2* (48), **(C)** *Trial design N3* (57), **(D)** *Trial design N4* (55), **(E)** *Trial design N5* (58), **(F1,F2)** *Trial design N6* (58) subject-Sch, **(G1,G2)** *Trial design N6* (58) subject-C, and **(H1,H2)** *Trial design N7* (27). See the Method section for trial settings.

**Figure 6:**
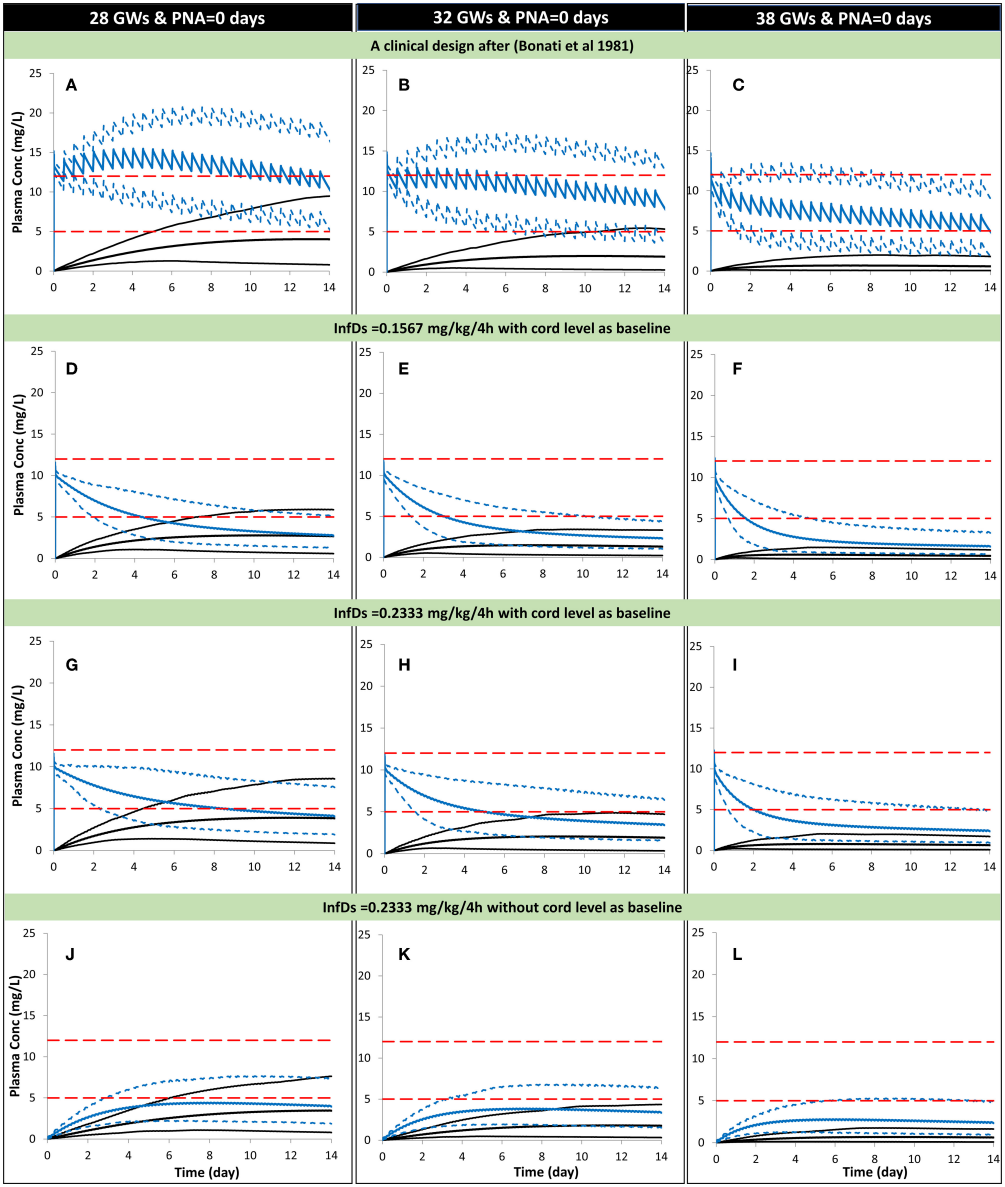
Predicted mean (5th−95th percentiles) neonatal theophylline (and formed caffeine) concentration during the first 2 weeks of life with different gestational weeks. Predicted scenarios: The first three plots show, respectively, theophylline exposure in neonates at birth using a dosage of 30-min infusion of 5 mg/kg as a loading dose followed by 1 h-infusion of 1.1 mg/kg/12 h in 28 **(A)**, 32 **(B)**, and 38 **(C)** GWs according to a clinical study (28). Exposure after oral administration of the calculated theophylline infant dose using milk C_avg,ss_ are shown for 28 **(D)**, 32 **(E)**, and 38 **(F)** GW considering the cord level at birth as a baseline. Exposure after oral administration of the calculated theophylline infant dose using milk C_max,ss_ are shown for 28 **(G)**, 32 **(H)**, and 38 **(I)** GW considering the cord level at birth as a baseline. Exposure after oral administration of the calculated theophylline infant dose using milk C_max,ss_ are shown for 28 **(J)**, 32 **(K)**, and 38 **(L)** GW without considering the cord level at birth as a baseline. **(A–D)** *Trial design N8*, **(D–F)** *Trial design N9*, **(G–I)** *Trial design N10*, and plo **(J–L)** *Trial design N11*. See the Method section for trial settings. Dashed horizontal lines represent the theophylline therapeutic window for apnea. InfDs, infant dose predicted using the lactation model; GWs, gestational weeks; PNA, postnatal age. The lowest profiles in each plot represent the mean (5th−95th percentiles) for the formed caffeine.

The preterm PBPK model replicated the observed exposure of theophylline and its metabolite caffeine after i.v. and oral doses (Figure 5). Figure 6 shows the simulation results for systemic theophylline (and formed caffeine) exposure in neonates (of different gestational weeks) compared to the suggested theophylline therapeutic window for apnea (60). The doses in milk were divided equally into 6 daily oral doses to resemble a 4-h frequency pattern of breastfeeding after birth, i.e., C_avg,ss_ dose was 0.157 mg/kg/4 h and C_max,ss_ dose was 0.233 mg/kg/4 h. The concentration in the cord at birth was included as a baseline exposure to mimic the real clinical situation. Simulations were performed for a duration of 2 weeks. Within the PBPK model, the total doses and physiological parameters (including changes in body weight) were continually updated to account for neonatal growth and development over the 14-day simulation period. Predicted systemic exposure in neonates at birth with either 28, 32, or 38 GWs is shown in Figure 6.

In a preterm population of 32 GW at birth the predicted mean concentration of theophylline decreased from 7.7 (6–9.3) to 2.3 (1–4.3) mg/L [mean (5th−95th percentiles)] between day 1 and day 14 in subjects receiving the repeated C_avg,ss_ dose. At the higher C_max,ss_ dose the concentration of theophylline was simulated to decrease from 8.1 (6.3–9.8) to 3.4 (1.5–6.4) mg/L between day 1 and day 14.

## Discussion

This work presented in this manuscript describes the use of a PBPK framework approach to describe the pharmacokinetics of a model drug (theophylline) during the perinatal period. Drug concentrations were predicted in the mother, the unborn fetus, and the neonatal subject post-delivery (Figure 1). Theophylline was chosen as a model drug based on the amount of data available for verification of model predictions and because it is often used to treat neonatal apnea.

The perinatal PBPK model presented here adequately predicted the observed exposure and kinetics of theophylline in non-pregnant, pregnant, lactating, and preterm populations. This type of simulation approach using a PBPK model allows drug concentrations to be predicted in populations of individuals that are otherwise difficult to study and also offers the possibility of supplementing sparse samples obtained in vulnerable populations with additional information, facilitating the design of future studies and also developing or refining hypotheses for future testing.

The PBPK model predictions for theophylline PK in the non-pregnant population after oral and intravenous administrations were in good agreement with observed values in different studies of variable dosing levels (Figure 2 and Table 1). All PK parameters were within 2-fold of the observed values and predicted 5th and 95th percentiles for the systemic exposure in plasma including the observed concentration vs. time profiles. The predicted bioavailability in the model was 0.96 ± 0.03, which is in agreement with the observed value of 0.99 ± 0.02 (19).

The reduction of CYP1A2 activity that occurs during pregnancy (3, 61) together with the increase in renal function and CYP2E1 activity considered within the pregnancy PBPK model adequately described the changes in theophylline pharmacokinetics during the whole gestational period (Figure 3). The CYP2E1 activity was calculated to increase by 1.8-fold at term compared to the pre-pregnancy values. The values calculated in this study are in agreement with the observed 1.87-fold (62) and 1.79-fold (63) increase in acetaminophen clearance to its glutathione-derived conjugate in pregnant women at term. Acetaminophen glutathione conjugate formation has been proposed as a marker metabolic pathway for CYP2E1 activity.

The progression of gestation dynamically changes the contribution of the different elimination pathways to the overall clearance of theophylline (see Supplementary Figure 2). The pregnancy PBPK model predicted an overall 40% reduction in theophylline clearance at term from the non-pregnant clearance, which agrees with clinical data (Table 1). A potential weakness of the developed PBPK model is that no physiological changes to intestinal physiology during pregnancy were considered, however as there was good agreement between the predicted and observed theophylline concentrations at different gestational weeks Any pregnancy-related changes in intestinal physiology or enzyme levels appear to have minimal impact on theophylline pharmacokinetics. This can be partially explained by the minimal gut metabolism, Fg ~1, and almost complete bioavailability, F ~1.0, observed in non-pregnant subjects (19).

Figure 3 shows that the incorporation of the theophylline transplacental clearance from an *ex vivo* experiment resulted in the adequate prediction of the theophylline umbilical cord level when compared to the observed values. The theophylline PBPK model predicted cord/maternal ratio based on AUC at steady-state is 1.0 (5th−95th percentiles: 0.86–1.2) and ranges from 0.8–1.3 in different virtual individuals. This is in line with the clinically reported ratio of unity from sparse data observed in 2 (64), 6 (65), 10 (47), and 12 (46) subjects at delivery. Because of the high transplacental passage of theophylline, toxicity in neonates at birth has been reported after multiple doses to the mother before delivery (66, 67).

Lactating mothers sometimes need to take medication whilst breastfeeding and are therefore confronted with a difficult choice to either discontinue breastfeeding or stop their medication to avoid potentially harmful effects on their breastfed children. The lack, or even the poor quality, of information about drug safety during lactation can cause confusion, which can result in the early cessation of breastfeeding (68). Different drugs (or environmental chemicals) will carry different levels of risks to the breastfed infant (69). Although these risks should not be exaggerated, since neonates and infants in most cases receive a much lower dose in breast milk, compared to the known safe dose of the same drug administered to them in clinical pediatric wards (70), there is still a possibility that some drugs could be harmful to breastfed children or may have not yet adequately been studied meaning that precautions should be taken. PBPK modeling offers one potential approach to address some of these questions. Another important aspect in this regard is to have quantitative information on drug exposure in breast milk both to verify the performance of PBPK models with a wider spectrum of compounds but also to allow the development of better algorithms with a wider domain of applicability to predict milk concentrations for drugs where quantitative measurements are lacking. Together the PBPK modeling approaches and quantitative information or prediction of milk concentrations have the potential to reduce the confusion and anxiety that lactating women may experience when making decisions about whether or not to take medication during breastfeeding, especially for those nursing mothers who are on chronic medication.

The reported theophylline elimination rate constant for the milk was 0.122 ± 0.051 h^−1^, which was not significantly different from that in plasma 0.123 ± 0.041 h^−1^ (52) indicating rapid equilibrium between the two matrices. Two different methods were used to predict the theophylline M/P ratio. These methods resulted in different predicted M/P ratios (0.49 for Model I and 0.87 for Model II; see Supplementary Figure 3). As a pragmatic approach the average M/P ratio of the 2 values, i.e., 0.68 (5%CV) was used to calculate the dose in the neonatal PBPK simulation. Studies with a larger set of compounds are needed to determine which of the methods or whether using an average value from the two methods would be the most appropriate approach for *a priori* prediction for a drug where the M/P ratio is unknown.

The average value for the M/P ratio used in the simulations reported here is in line with reported values in the literature from several different sources. For instance, an observed mean M/P ratio of 0.79 was reported by Gardner et al. (45), a range of values 0.6–0.89 was reported by Reinhardt et al. (52), an observed mean value of 0.67 was reported by Stec et al. (51), and a value of 0.57 ± 0.14 was reported by Oo et al. (71). The calculated absolute infant dose based on milk C_max,ss_ was 1.4 ± 0.5 (5th−95th percentiles: 0.74–2.1) mg/kg/day. This dose is less than the loading intravenous dose of 5 mg/kg theophylline (28, 58, 60) and in line with the intravenous maintenance, dose using a 1-h infusion of 1.1 mg/kg/12 h (28) to treat apnea of prematurity. The individual maintenance dose is titrated to individual needs and some neonates may need maintenance doses as high as 4.4 mg/kg per day (60). Although the calculated dose from milk exposure is in line with the maintenance infusion dose used for apnea treatment, accumulation of theophylline can occur, especially in premature infants due to the lower capacity of their CYP1A2 clearance pathway, which can lead to adverse effects.

The neonatal PBPK model adequately described theophylline and its formed metabolite, caffeine, exposure after intravenous infusion, and after oral administration of theophylline (Figure 5). In contrast to a previously published theophylline PBPK in preterm neonates (72), the current model applied dynamic change in growth physiology, and utilized ontogeny functions to describe the maturation of CYP1A2 and CYP2E1 enzymes. In addition, the performance of the theophylline PBPK model in neonatal subjects was verified with multiple clinical studies. Table 1 shows the predicted clearance in preterm subjects. The predicted values agreed with the observed values after intravenous (28, 57) and oral administration of theophylline (58). The contribution of the different elimination pathways to the clearance of theophylline at different gestational and postnatal ages is shown in Supplementary Figure 5.

The higher concentrations of caffeine compared to theophylline after the cessation of theophylline therapy were due to the (~2- to 4-fold) slower elimination and continued formation of caffeine as theophylline was cleared (27, 58, 73). In the developed preterm PBPK model there is a futile recycling process occurring whereby theophylline is methylated to form caffeine that is subsequently demethylated to theophylline by CYP1A2 and CYP2E1.

The theophylline plasma levels in neonates (32GW) were simulated using the PBPK model with the dose based on the milk C_avg,ss_, and C_max,ss_ and the assumption that babies ingest about 150 mL/kg/day and that feeding is split over 6 sessions 4-h apart. Under these assumptions and using a bolus dose to set the initial concentration in neonatal plasma to be the same as the concentration in the cord blood at birth the theophylline concentrations were shown to remain within the therapeutic range for 2–4 days post-birth. The length of time the concentration stayed in the therapeutic range varied with the gestational age due to the elimination mechanisms of theophylline being immature at birth and varying with the gestational age of the neonates.

Within the PBPK model for the neonate subjects, the conversion of theophylline to caffeine was considered. But the simulations showed that the caffeine levels predicted in the neonatal plasma of individuals who ingested theophylline *via* the milk were too low to exert a significant pharmacological effect. At therapeutic doses such as those used to treat apnea in very premature babies, the caffeine concentrations were at a level that may contribute to the pharmacological effect of theophylline (Figure 6A).

The infant's daily dose based on milk C_max,ss_ with an administered volume of 150 mL of milk/day per kg infant bodyweight being ingested was used as a fairly conservative scenario in this analysis. In reality, the nursing mother may not start breastfeeding straight after birth or may not produce 150 mL of milk in the first few days post-partum. As the therapeutic window of theophylline is not well-defined for premature apnea the recommended range defined by Jones and Baillie was used for comparison with the simulated data in this study (60).

The theophylline level in milk or in the neonatal circulation can also be influenced by other intrinsic or extrinsic factors, such as co-medication, and/or disease. The maturation of theophylline elimination with age showed that the theophylline levels decline with age more rapidly in older newborns at 38 GWs compared with those born at 28 GWs (Figure 6).

While this work shows a case study of the application of the PBPK model during the perinatal period, there are limitations to the study. No attempt was made to include fetal metabolism in the maternal-fetal PBPK model due to a lack of data to parameterize the fetal metabolism model and verify the performance of the model. The contribution of fetal metabolism to the overall drug elimination is expected to be small for three reasons; (1) the transplacental passage of theophylline is high (cord/maternal ratio is ~1) (2) the metabolism of theophylline in fetal liver explants (12–20 GWs) was slow ~1.25 nmol/day (74), and (3) the reported absence of CYP1A2 protein in the fetal and neonatal livers (75). Theophylline PK data in the fetoplacental unit are limited to a few observations during delivery (46–48). There are limitations with the available data in that the dosing history prior to the studied doses at a steady state was not available during pregnancy and lactation. This makes accurate simulation of the clinical studies more difficult as some assumptions need to be made, i.e., the studied dosing regimen at steady-state and formulation was assumed since initiation of therapy (45, 46). In some studies where different subjects were studied after receiving a different number of doses and this information was not available, an average number of doses was assumed (47). The issue with incomplete dosing information was also encountered in some of the lactation studies, where three subjects were studied but only the dose range was reported (51). In another study, only a single profile from the 12 studied subjects was reported for the plasma and milk concentration profiles (52) making a meaningful comparison of the simulated and observed data difficult. In the lactation studies, information on milk pH and fat content were not available necessitating an assumption in the PBPK model that the milk composition was the same as that of mature milk. The lactation model used in this work assumes that a rapid equilibrium of drug concentrations exists between plasma and milk. This assumption was sufficient to describe theophylline exposure in the milk, but for other (more lipophilic) drugs the time course of drug concentrations in plasma and milk may not increase or decrease in parallel, and in these cases, the milk profiles cannot be explained with an assumption of rapid equilibration between the two phases.

The simulations in adult subjects were performed using a mechanistic absorption model (76). Due to a lack of detailed intestinal physiology in the preterm neonate, the same approach could not be used to model the absorption of theophylline in the neonate subjects. Therefore, a first-order absorption model was used in the preterm PBPK model. Although verification of the first-order absorption model in preterm studies was performed it would be preferable to have used the same absorption model for all of the different scenarios that were simulated.

The available preterm PK data were reported without the details of the postnatal and gestational ages for individual subjects and usually subjects with different gestational ages were lumped together in the reported data. This makes it difficult to simulate the studies with matched subjects (in terms of demographics). While there are multiple unpowered studies that have attempted to investigate theophylline pharmacokinetics in preterm individuals, to the best of our knowledge, longitudinal assessments of drug levels in this population after birth with or without considering the contribution of ingested drugs in the milk have not been reported in the literature. A further complication is that the observed data in the literature are usually reported from preterm subjects under treatment and as such the exposure information may also be influenced by co-morbidity and/or comedication factors. For example, the theophylline half-life was reported to be 39.4 h in a group of individuals co-dosed with betamethasone compared to a half-life of 61 h in the control group in two age-matched preterm groups of 29.6 gestational weeks and 1–3 days. Even after a few weeks of treatment, the theophylline half-life remained higher in the control group compared with the betamethasone treatment group, 31 vs. 19 h (77).

## Conclusion

A PBPK approach was adopted to evaluate the pharmacokinetics of theophylline from the general population and at different gestational weeks throughout pregnancy as well as in the plasma and milk of lactating women and in plasma from neonatal subjects. Utilizing a PBPK approach in special populations reinforces the utility of PBPK to assess pharmacokinetics in clinical settings where clinical data are limited and can be used to improve study design in these vulnerable populations.

## Resource Identification Initiative

The Simcyp Simulator V21 (Simcyp, RRID:SCR_003944) was used for the assessment of theophylline pharmacokinetics using the PBPK approach.

## Data Availability Statement

The original contributions presented in the study are included in the article/Supplementary Material, further inquiries can be directed to the corresponding author/s.

## Author Contributions

KA collected the data, analyzed the data, and wrote the manuscript. IG and MJ reviewed the manuscript. All authors contributed to the article and approved the submitted version.

## Conflict of Interest

KA, IG, and MJ are paid employees of Certara UK Limited (Simcyp Division) and may hold shares in Certara.

## Publisher's Note

All claims expressed in this article are solely those of the authors and do not necessarily represent those of their affiliated organizations, or those of the publisher, the editors and the reviewers. Any product that may be evaluated in this article, or claim that may be made by its manufacturer, is not guaranteed or endorsed by the publisher.
