# Supplementary material for: Application of a Physiologically Based Pharmacokinetic Approach to Predict Theophylline Pharmacokinetics Using Virtual Non-Pregnant, Pregnant, Fetal, Breast-Feeding, and Neonatal Populations
Source: Front Pediatr. 2022 May 12;10:840710. doi: 10.3389/fped.2022.840710 (PMC9150776; doi:10.3389/fped.2022.840710)
Supplement: Supplementary file 1 [file Data_Sheet_1.docx]

Supplementary materials

Application of a Physiologically Based Pharmacokinetic Approach to Predict Theophylline Pharmacokinetics Using Virtual Non-Pregnant, Pregnant, Fetal, Breast-feeding, and Neonatal Populations

Khaled Abduljalil^1*^, Iain Gardner^1^, Masoud Jamei ^1^

^1^Certara UK Limited (Simcyp Division), Level 2-Acero, 1 Concourse Way, Sheffield, S1 2BJ, United Kingdom

Table 1. Theophylline PBPK model input parameters

| **Parameter** | **Value (unit)** | **Reference** |
| --- | --- | --- |
| Molecular Weight | 180.2 g/mol | (PubChem) |
| log P | -0.02 | (Hansch et al., 1995) |
| Compound Type | Ampholyte | (PubChem) |
| pKa 1 (acid), pKa2 (base) | 8.8, 0.99 | (PubChem) |
| B/P | 0.815 | (Habib et al., 1987) |
| Main plasma binding protein | Human serum albumin |  |
| Fraction unbound in plasma, fu | 0.5 | (Lombardo et al., 2004) |
| **Absorption** | Predicted |  |
| Absorption model option | ADAM (for adult)  First order model (for preterm) |  |
| Intrinsic Solubility | 1.976 mg/mL | Predicted from melting point 273^o^C (PubChem) according to Jain et al., (Jain and Yalkowsky, 2001) |
| Permeability (Peff, man) | 4.0673 x10^-4^ cm/s  (for adult and preterm) | Predicted using MechPeff model within the Simcyp Simulator V21 |
| **Distribution Model** |  |  |
| Distribution volume input type | Full PBPK |  |
| Vss | 0.37 L/kg | Predicted according to Rodgers & Rowland (Rodgers and Rowland, 2006) |
| Global tissue to plasma (Kp) Scalar | 1.2 | Optimised using Gisclon et al., (Gisclon et al., 1997) |
| **Elimination model** |  |  |
| Renal Clearance | 0.31 L/h | (Lo et al., 1989; Rasmussen et al., 1997) |
| Metabolism -option | Recombinant Enzyme kinetics |  |
| CYP1A2 (N1-demethylation) Vmax    CYP1A2 (N1-demethylation) Km | 2.47 pmol/min/mg protein  1080 uM | Optimised: Clint back calculated from CLpo from a meta-analysis of 8 studies.  (Ha et al., 1995) |
| CYP1A2 (N3-demethylation) Vmax  CYP1A2 (N3-demethylation) Km | 6 pmol/min/mg protein  377 uM | Optimised: see above.  (Ha et al., 1995) |
| CYP2D6 (N3-demethylation) Vmax  CYP2D6 (N3-demethylation) Km | 1.8 pmol/min/mg protein  6897 uM | Optimised: see above.  (Ha et al., 1995) |
| CYP1A2 (8-OH) Vmax  CYP1A2 (8-OH) Km | 4.11 pmol/min/mg protein  394 uM | Optimised: see above.  (Ha et al., 1995; Zhang and Kaminsky, 1995) |
| CYP2D6 (8-OH) Vmax  CYP2D6 (8-OH) Km | 4.68 pmol/min/mg protein  10709 uM | Optimised: see above.  (Ha et al., 1995; Zhang and Kaminsky, 1995) |
| CYP2E1 (8-OH) Vmax  CYP2E1 (8-OH) Km | 40.78 pmol/min/mg protein  16855 uM | Optimised: see above.  (Ha et al., 1995; Zhang and Kaminsky, 1995) |
| CYP3A4 (8-OH) Vmax  CYP3A4 (8-OH) Km | 0.4 pmol/min/mg protein  23393 uM | Optimised: see above.  (Zhang and Kaminsky, 1995) |
|  |  |  |
| **Specific parameters for the Fetoplacental model** | |  |
| Placental passive diffusion clearance CL_PD_ | 0.0071 L/h/mL placenta | (Omarini et al., 1993) |
| Fetal-to-amniotic clearance, CL_F-AF_ (=Fetal CL_R_+CL_Intramembraneous_) | 0.00776 L/h/kg fetal weight | (0.00359+0.00417) see Method section |
| Amniotic-to-fetal clearance, CL_AF-F_ (=Fetal swallowing + CL_Intramembraneous_) | 0.00893 L/h/kg fetal weight | (0.00476 + 0.00417) see Method section |
|  |  |  |
| **Specific parameters for the preterm model** | |  |
|  |  |  |
| Absorption: ka (user-input) | 1.0444 1/h (50% CV) | Simcyp Simulator V21 Prediction |
| Absorption: fa (user-input) | 0.8 (30%CV) | Optimized using preterm oral data (Elias-Jones et al., 1985) |
| Elimination: Caffeine formation CLint (Preterm only) | 0.04 (µL/min/mg protein) | Optimised using IV data (Bonati et al., 1981) |

Table 2. Caffeine PBPK model input parameters

| **Parameter** | **Value (unit)** | **Reference** |
| --- | --- | --- |
| Molecular Weight | 194.2. g/mol | (PubChem) |
| log P | -0.07 | (Hansch et al., 1995) |
| Compound Type | Monoprotic base |  |
| pKa 1 | 1.05 | Unpublished measured value |
| B/P | 0.977 | Unpublished measured value |
| Main plasma binding protein | Human serum albumin |  |
| Fraction unbound in plasma, fu | 0.68 | (Lelo et al., 1986) |
|  |  |  |
| **Distribution Model** |  |  |
| Distribution volume input type | Full PBPK |  |
| Vss | 0.416 L/kg | Predicted according to Rodgers & Rowland (Rodgers and Rowland, 2006) |
| Global tissue to plasma (Kp) Scalar | 1.7 | Adjusted to recover (Charles et al., 2008) see (Abduljalil et al., 2020) |
| **Elimination model** |  |  |
| Renal Clearance | 0.038 L/h | (Birkett and Miners, 1991) |
| Metabolism -option | Recombinant Enzyme kinetics |  |
| CYP1A2 (N1-demethylation) Vmax  CYP1A2 (N1-demethylation) Km | 0.56 pmol/min/mg protein  157 uM | (Bloomer et al., 1995; Ha et al., 1996) |
| CYP2E1 (N1-demethylation) Vmax  CYP2E1 (N1-demethylation) Km | 0.03 pmol/min/mg protein  1411 uM | (Ha et al., 1996) |
| CYP1A2 (N3-demethylation) Vmax  CYP1A2 (N3-demethylation) Km | 13.6 pmol/min/mg protein  300 uM | (Ha et al., 1996) |
| CYP1A2 (N7-demethylation) Vmax  CYP1A2 (N7-demethylation) Km | 0.21 pmol/min/mg protein  245 uM | (Ha et al., 1996) |
| CYP2E1 (N7-demethylation) Vmax  CYP2E1 (N7-demethylation) Km | 0.02 pmol/min/mg protein  823 uM | (Ha et al., 1996) |
| CYP1A2 (8-OH) Vmax  CYP1A2 (8-OH) Km | 0.36 pmol/min/mg protein  265 uM | (Ha et al., 1996) |
| CYP2E1 (8-OH) Vmax  CYP2E1 (8-OH) Km | 0.18 pmol/min/mg protein  1019 uM | (Ha et al., 1996) |
| CYP3A4 (8-OH) Vmax  CYP3A4 (8-OH) Km | 1.8 pmol/min/mg protein  45080 uM | (Ha et al., 1996) |

**Supplementary Figure 1.** Gestational-dependent CYP2E1 activity. The change in CYP2E1 activity during pregnancy was estimated using clinical unbound oral clearance data of theophylline at different gestational weeks (Gardner et al., 1987) and those converted to unbound from total clearance (Carter et al., 1986) using the predicted protein binding at each gestational week by the model. A complete bioavailability was assumed, i.e. F=1 in line with the observed data reported by Hendeles et al., (Hendeles et al., 1977). Oral theophylline clearance with (middle) and without (bottom) accounting for CYP2E1 changes during pregnancy. Dashed lines are the predicted 5^th^ and 95^th^ percentiles around the predicted mean (solid line) from the PBPK simulations. Observations: circles (Carter et al., 1986), squares (Gardner et al., 1987).


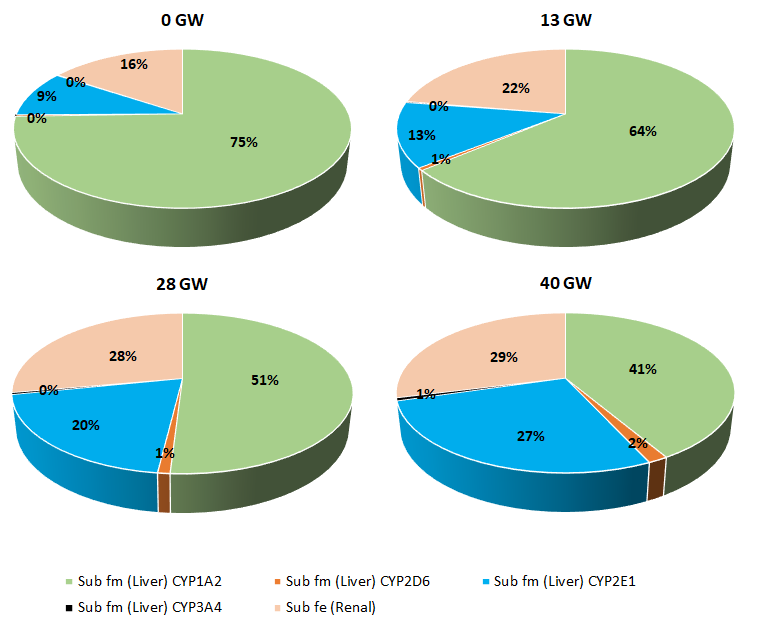


**Supplementary Figure 2.** Contributions of different elimination pathways to the total clearance of theophylline during pregnancy

**Supplementary Figure 3.** Plasma and milk conc profile predicted using Phase-Distribution (Model I) and Log-Distribution (Model II) models. Circles are observed data from Stec et al., (Stec et al., 1980), Gardner et al., (Gardner et al., 1987), and Reinhardt et al. (Reinhardt et al., 1983). Solid lines are predicted mean, Dashed lines are predicted 5^th^ and 95^th^ percentiles.

**Supplementary Figure 4.** Sensitivity analysis performed for intestinal ontogeny (varied between no ontogeny, x1 and 30-fold increase, x30) of the generic enzyme representing xanthine methylation (observed mean data (Elias-Jones 1985).

**
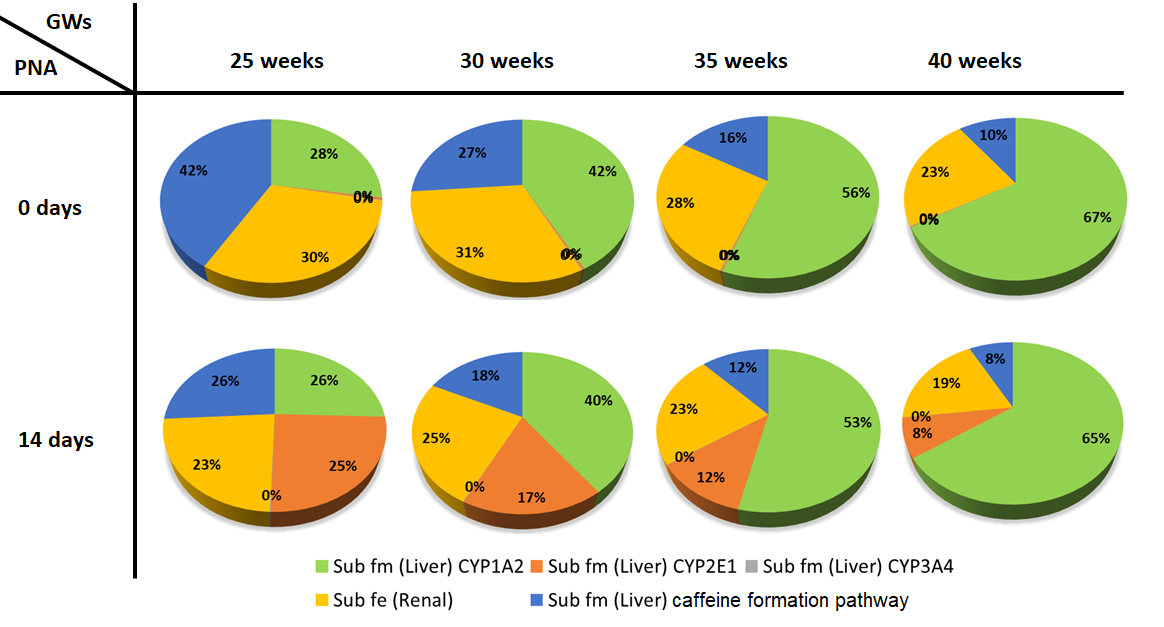
Supplementary Figure 5.** Contributions of different elimination pathways to the total clearance of theophylline in preterm neonates.

**References**

Abduljalil, K., Pan, X., Pansari, A., Jamei, M., and Johnson, T.N. (2020). Preterm Physiologically Based Pharmacokinetic Model. Part II: Applications of the Model to Predict Drug Pharmacokinetics in the Preterm Population. *Clin Pharmacokinet* 59(4)**,** 501-518. doi: 10.1007/s40262-019-00827-4.

Birkett, D.J., and Miners, J.O. (1991). Caffeine renal clearance and urine caffeine concentrations during steady state dosing. Implications for monitoring caffeine intake during sports events. *Br J Clin Pharmacol* 31(4)**,** 405-408. doi: 10.1111/j.1365-2125.1991.tb05553.x.

Bloomer, J.C., Clarke, S.E., and Chenery, R.J. (1995). Determination of P4501A2 activity in human liver microsomes using [3-14C-methyl]caffeine. *Xenobiotica* 25(9)**,** 917-927. doi: 10.3109/00498259509046663.

Bonati, M., Latini, R., Marra, G., Assael, B.M., and Parini, R. (1981). Theophylline metabolism during the first month of life and development. *Pediatr Res* 15(4 Pt 1)**,** 304-308. doi: 10.1203/00006450-198104000-00003.

Carter, B.L., Driscoll, C.E., and Smith, G.D. (1986). Theophylline clearance during pregnancy. *Obstet Gynecol* 68(4)**,** 555-559.

Charles, B.G., Townsend, S.R., Steer, P.A., Flenady, V.J., Gray, P.H., and Shearman, A. (2008). Caffeine citrate treatment for extremely premature infants with apnea: population pharmacokinetics, absolute bioavailability, and implications for therapeutic drug monitoring. *Ther Drug Monit* 30(6)**,** 709-716. doi: 10.1097/FTD.0b013e3181898b6f.

Elias-Jones, A.C., Dhillon, S., and Greenough, A. (1985). The efficacy of oral theophylline in ventilated premature infants. *Early Hum Dev* 12(1)**,** 9-14. doi: 10.1016/0378-3782(85)90131-8.

Gardner, M.J., Schatz, M., Cousins, L., Zeiger, R., Middleton, E., and Jusko, W.J. (1987). Longitudinal effects of pregnancy on the pharmacokinetics of theophylline. *Eur J Clin Pharmacol* 32(3)**,** 289-295. doi: 10.1007/BF00607577.

Gisclon, L.G., Curtin, C.R., Fowler, C.L., Williams, R.R., Hafkin, B., and Natarajan, J. (1997). Absence of a pharmacokinetic interaction between intravenous theophylline and orally administered levofloxacin. *J Clin Pharmacol* 37(8)**,** 744-750. doi: 10.1002/j.1552-4604.1997.tb04362.x.

Ha, H.R., Chen, J., Freiburghaus, A.U., and Follath, F. (1995). Metabolism of theophylline by cDNA-expressed human cytochromes P-450. *Br J Clin Pharmacol* 39(3)**,** 321-326. doi: 10.1111/j.1365-2125.1995.tb04455.x.

Ha, H.R., Chen, J., Krahenbuhl, S., and Follath, F. (1996). Biotransformation of caffeine by cDNA-expressed human cytochromes P-450. *Eur J Clin Pharmacol* 49(4)**,** 309-315. doi: 10.1007/BF00226333.

Habib, M.P., Schifman, R.B., Shon, B.Y., Fiastro, J.F., and Campbell, S.C. (1987). Evaluation of whole blood theophylline enzyme immunochromatography assay. *Chest* 92(1)**,** 129-131. doi: 10.1378/chest.92.1.129.

Hansch, C., Hoekman, D., Leo, A., Zhang, L., and Li, P. (1995). The expanding role of quantitative structure-activity relationships (QSAR) in toxicology. *Toxicol Lett* 79(1-3)**,** 45-53. doi: 10.1016/0378-4274(95)03356-p.

Hendeles, L., Weinberger, M., and Bighley, L. (1977). Absolute bioavailability of oral theophylline. *Am J Hosp Pharm* 34(5)**,** 525-527.

Jain, N., and Yalkowsky, S.H. (2001). Estimation of the aqueous solubility I: application to organic nonelectrolytes. *J Pharm Sci* 90(2)**,** 234-252. doi: 10.1002/1520-6017(200102)90:2<234::aid-jps14>3.0.co;2-v.

Lelo, A., Birkett, D.J., Robson, R.A., and Miners, J.O. (1986). Comparative pharmacokinetics of caffeine and its primary demethylated metabolites paraxanthine, theobromine and theophylline in man. *Br J Clin Pharmacol* 22(2)**,** 177-182. doi: 10.1111/j.1365-2125.1986.tb05246.x.

Lo, K.F., Nation, L., and Sansom, L.N. (1989). Lack of effect of co-trimoxazole on the pharmacokinetics of orally administered theophylline. *Biopharm Drug Dispos* 10(6)**,** 573-580. doi: 10.1002/bdd.2510100606.

Lombardo, F., Obach, R.S., Shalaeva, M.Y., and Gao, F. (2004). Prediction of human volume of distribution values for neutral and basic drugs. 2. Extended data set and leave-class-out statistics. *J Med Chem* 47(5)**,** 1242-1250. doi: 10.1021/jm030408h.

Omarini, D., Barzago, M.M., Bortolotti, A., Lucchini, G., Stellari, F., Efrati, S., et al. (1993). Placental transfer of theophylline in an in vitro closed perfusion system of human placenta isolated lobule. *Eur J Drug Metab Pharmacokinet* 18(4)**,** 369-374. doi: 10.1007/BF03190187.

PubChem *Theophylline.* [Online]. PubChem. Available: <https://pubchem.ncbi.nlm.nih.gov/compound/Theophylline> [Accessed 07/12/2021].

Rasmussen, B.B., Jeppesen, U., Gaist, D., and Brosen, K. (1997). Griseofulvin and fluvoxamine interactions with the metabolism of theophylline. *Ther Drug Monit* 19(1)**,** 56-62. doi: 10.1097/00007691-199702000-00010.

Reinhardt, D., Richter, O., and Brandenburg, G. (1983). [Pharmacokinetics of drugs from the breast-feeding mother passing into the body of the infant, using theophylline as an example]. *Monatsschr Kinderheilkd* 131(2)**,** 66-70.

Rodgers, T., and Rowland, M. (2006). Physiologically based pharmacokinetic modelling 2: predicting the tissue distribution of acids, very weak bases, neutrals and zwitterions. *J Pharm Sci* 95(6)**,** 1238-1257. doi: 10.1002/jps.20502.

Stec, G.P., Greenberger, P., Ruo, T.I., Henthorn, T., Morita, Y., Atkinson, A.J., Jr., et al. (1980). Kinetics of theophylline transfer to breast milk. *Clin Pharmacol Ther* 28(3)**,** 404-408. doi: 10.1038/clpt.1980.180.

Zhang, Z.Y., and Kaminsky, L.S. (1995). Characterization of human cytochromes P450 involved in theophylline 8-hydroxylation. *Biochem Pharmacol* 50(2)**,** 205-211. doi: 10.1016/0006-2952(95)00120-o.
